# Epidemiology of severe mental illness in Hunan province in central China during 2014-2015: A multistage cross-sectional study

**DOI:** 10.1371/journal.pone.0188312

**Published:** 2017-11-29

**Authors:** Dongxin Wang, Jing Ma, Lihong Tan, Yan Chen, Xiaosong Li, Xuefei Tian, Xuhui Zhou, Xuejun Liu

**Affiliations:** 1 Hunan Institute of Mental Health, the Brain Hospital of Hunan Provincial, Changsha, Hunan, China; 2 Department of disease control, Health and family planning commission of Hunan province, Changsha, Hunan, China; University of Texas Health Science Center at San Antonio Cancer Therapy and Research Center at Houston, UNITED STATES

## Abstract

**Objective:**

Severe mental illness (SMI) represents major social and public health problem in China, especially in low- or middle-income regions. We aim to assess the prevalence and distribution of SMI in Hunan province in central China.

**Methods:**

Multistage stratified random sampling methods were used to select qualified subjects in 123 districts and counties in Hunan province. 89465 individuals were randomly identified, and 72999 (81.6%) completed the supplemental 12-Item General Health Questionnaire (GHQ-12) and Cue questionnaire of psychiatric abnormal behaviors. 6082 suspected individuals having high or moderate risk, or psychiatric cues, were administered the Structured Clinical Interview for DSM-IV Axis I disorders (SCID-I) by psychiatrists.

**Results:**

720 respondents were definitely diagnosed as SMI. The 1-month and lifetime prevalence was 9.35‰ and 10.10‰, respectively. The most frequent SMI was schizophrenia, followed by bipolar disorder, intellectual disability, epileptic mental disorder, paranoid psychosis and schizoaffective disorders, with 1-month prevalence ranging from 0.11‰ to 6.50‰ and lifetime prevalence ranging from 0.24‰ to 6.86‰. Multivariate logistic regression analysis revealed that lower education, farmer occupation, retirees or jobless/unemployed, unmarried or divorced and age of 30–64 years old were major factors that associated with the increased risk of SMI. In addition, only 33.3% of 528 patients who completed questionnaire sought help in psychiatric institutions, and up to 51.7% of 720 patients were not referred to the SMI management system in Hunan province.

**Conclusions:**

These findings provided a large-scale prevalence data of SMI in a provincial sample of China. The psychiatric disorders brought economical and psychological burden for family and society, which may shed light on the significance of scaling up province-wide mental health service and strengthening the SMI management.

## Introduction

Severe mental illness (SMI) is believed to account for a large portion of disease burden and is the leading cause of disability worldwide [1, 2]. Violence and self-harm behaviors are more common in persons with SMI than in the general population, and cause great public health concerns and challenge [3–5]. More attention should be paid to the prevention and control of the disease. SMI mainly includes schizophrenia, bipolar disorder, schizoaffective disorders, major depression, alcohol induced disorder etc. [6]. The psychiatric surveys of the general population have been widely carried out to estimate the prevalence in many areas, such as Britain, Australia, Netherlands, Mexico and USA. Mental disorders are highly prevalent in most countries throughout the world [7–13]. However, the national estimates of psychiatric morbidity from other countries were inappropriate to import as references for Chinese population. Perception of the SMI prevalence, impact and management in China are necessary to scale up mental health service and to make nation-wide mental health policy.

Since the first programme for treatment and management of SMI (“Central Government Support for the Local Management and Treatment of Severe Mental Illnesses Project”, 686 Project), was launched in 2004 by the Chinese government. More and more attentation has been focused on the treatment and management of the disease. SMI has been generally recognized as public health issues since 2009. The psychiatric epidemiological surveys have been therefore widely conducted to estimate the prevalence and distribution of mental illness in different areas of China [14–17]. Hunan province is an agricultural region located in the south of the Yangtze River. It is an intermediate-economically developed province. As for the Hunan province, only the two large-scale epidemiological surveys in 1982 (n = 38,136) and 1993 (n = 19 233) were involved. The time-point prevalence of SMI (including schizophrenia, mental retardationintellectual disability, severe affective psychosis, alcohol and drug dependence) were estimated to be 9.10‰ and 11.2‰ [13, 14]. No large scale epidemiological surveys focusing on SMI have been carried out since then. Schizophrenia, paranoid psychosis, bipolar disorder, schizoaffective disorders, epileptic mental disorder and intellectual disability are recognized as the major SMI in China according to the Standards for the Management and Treatment of Severe Mental Disorders (http://www.nhfpc.gov.cn/mohbgt/s9514/200911/44384.shtml). In view of their public health consequences, these six diseases have been managed in a unified manner using the uniform and standardized management strategy. Our study was therefore launched as the first large-scale severe psychiatric survey in Hunan province in central China, to produce representative data regarding to these severe psychiatric diseases. The survey reported here was to investigate (1) the prevalence rate of SMI in the population of Hunan province, China; (2) the socio-demographic and geographical distribution characteristics of prevalence; (3) the family-society impact and danger level of SMI; (4) the mental health service utilization and management status of individuals with SMI. Our study may not only provide updated epidemiological data on the major SMI in Hunan province of China, but also help to evaluate the management system used nationally.

## Materials and methods

### Survey design and participants

The survey was conducted in Hunan province from March, 2014 to July, 2015. Hunan province is a middle-income region with the largest population (about 67.37 millions) in central China. It consists of 14 cities and 123 counties. The multistage stratified random sampling methods were used to identify 123 counties and districts as sampling sites in 14 cities of Hunan province. A total of 150 households were sampled from each county and villagey of 123 counties. A computer-assisted interviewing (CAI) methodology was used in the face-to-face household survey as previous study reported [18]. All the electronic questionnaires of field survey were produced through interview expert software (Shanghai Nankang Technology Co., Ltd, China).

The target population was the permanent residents aged 15 years or older. Individuals met one of the following criteria were also included: 1) Foreign population live here for at least 6 months during the last 12 months; 2) Local residents leaving the area because of hospitalization, rehabilitation mental disorders, or imprisonment. Local registered residents who left the community for 1 or more years were excluded from the study.

The survey was mainly undertaken by the Health Planning Commission of Hunan Province, the Brain Hospital of Hunan Province, and the Hunan provincial Center for Disease Control and Prevention, the XiangYa School of Public Health and the Second XiangYa Hospital of Central South University.

Informed consent was obtained before the interview. All the design schemes and procedures were approved by the Ethics Committee of the Brain Hospital of Hunan Province & the Second People’s Hospital of Hunan Province. Respondents were interviewed in private at their places of residence.

### Survey content, survey tools and survey procedures

This prevalence estimation focused on schizophrenia, paranoid psychosis, bipolar disorder, schizoaffective disorders, epileptic mental disorder and intellectual disability, as the major SMI according to the Standards for the Management and Treatment of Severe Mental Disorders published in 2012 (http://www.gov.cn/gzdt/att/att/site1/20120412/1c6f6506c5d510f1076d01.pdf). In view of their public health consequences, these six kinds of SMI were also emphasized by National Health and Family Planning Commission.

**Fig 1:**
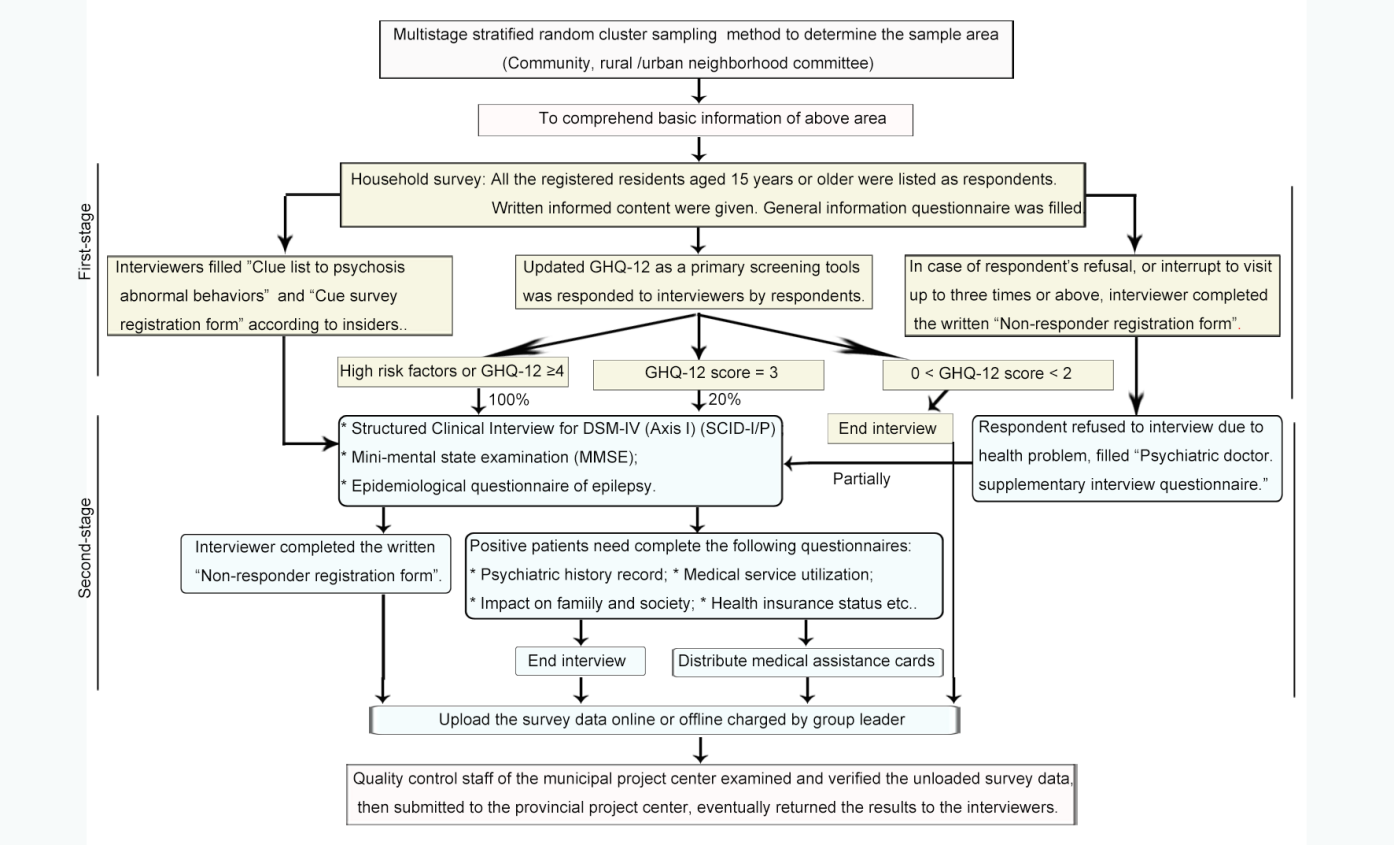
The flowchart of the sampling procedure. GHQ-12, the supplemental 12-Item General Health Questionnaire; CQPAB, Cue questionnaire of psychotic abnormal behaviors.

The flowchart of the sampling procedure is shown in Fig 1. Firstly, the suspected respondents with psychotic symptoms were selected from the whole sample population using supplemental 12-Item General Health Questionnaire (GHQ-12) [11, 14] and Cue questionnaire of psychotic abnormal behaviors (CQPAB) [19]. The interviews were completed by qualified investigators that leaded by 490 experts from Hunan provincial Center of Disease Control and Prevention during February 26 to March 31, 2015. GHQ-12 is an international valid screening instrument for mental disorders, with the advantage of simplicity, convenience and time-saving. GHQ score has been suggested to provide a rough indication of psychiatric problems for the whole respondents [20, 21]. To reduce the number of individuals lost to follow-up, GHQ-12 was mostly answered by respondents themselves, and partially by their spouse or children with close links to them. CQPAB is an 11-item screening questionnaire for abnormal behaviors (including suicide, self mutilation, previous mental hospitalization, being locked up at home, often babble, etc.). It was recommended according to the Standards for the Management and Treatment of Severe Mental Disorders developed by the Mental Health Division of the Ministry of Health of China (http://www.gov.cn/gzdt/att/att/site1/20120412/1c6f6506c5d510f1076d01.pdf). The questionnaire was answered by the village chief, neighborhood committee director, property management personnel, or neighbors etc. All respondents were classified into three risk strata for mental disorder according to the GHQ results: low (0–1 score), moderate (2–3 scores) and high risk (≥4 scores). Individuals at high and moderate risk strata or with abnormal behaviors according to CQPAB would be assigned to complete further diagnostic assessment of mental disorders. Individuals at low risk strata and with normal behaviors would be deprived of the interview.

Next, the suspected respondents completed the second-stage diagnosis during April 20 to May 30, 2015. These interviews were administered by appropriately 200 clinical psychologists after training. The diagnostic criteria for SMI or mental disorders were based on the Structured Clinical Interview for DSM-IV Axis I disorders (SCID-I) manual. SCID-I is a diagnostic instrument for clinical psychiatrists or trained mental health professionals who are familiar with the DSM system to make psychiatric diagnoses via an interviewing process [22, 23]. SCID as gold standard diagnostic tool has undergone several revisions. DSM-IV diagnostic categories and criteria were widely used in China for basic research and clinical application. Our semi-structured, psychiatrist-administered interview allowed the SCID questions about symptoms to be rephrased when respondents were confused [14].

Mini-mental state examination (MMSE) is the primary scale to assess cognitive impairment for respondents with longstanding cognitive or memory problems or track changes in cognitive function over time [24, 25]. In our survey, a culturally adapted Chinese version of MMSE [26] was used to assist in the diagnosis of intellectual disability after SCID-I assessment. MMSE was not recommended to make a definite diagnosis of dementia based on total MMSE scores alone [27, 28]. The epidemiological questionnaire of epilepsy was therefore used to assess the prevalence of epilepsy among the patients with mental disorders based on the WHO screening questionnaires [29–31]. The epilepsy questionnaire is an epidemiological survey recommended by the China Association Against Epilepsy: (G31) epilepsy. (uncontrollable shaking the arms or legs, fall suddenly and change colour in the face, lose consciousness, fall unconsciously, fall and bite your tongue or lose control of your bladder, shake or tremble in one arm or leg or in the face, lose contact with the surroundings and experience abnormal smells, been told have or have had convulsions, epilepsy or epileptic fits, etc.). The respondents with epilepsy were definitely diagnosed with epileptic mental disorder, while those without epilepsy were excluded by clinicians after SCID-I assessment.

All the respondents filled “Household general information questionnaire”. Individuals with a current diagnosis of SMI need to complete the following questionnaires: psychiatric history record, impact on family and society, danger assessment questionnaire, medical service utilization, reliability of the agent respondents etc. If the respondents could not fill the forms mentioned above, the written “Non-responder registration form” was asked to be completed by the interviewers. In case of respondent’s refusal or interruption up to three times or more, interviewer completed the written “Non-responder registration form”.

The impact on family and society were classified as: mild trouble, serious trouble, an accident, attempting suicide, automultilation and others. With regard to danger level of the SMI, danger assessment questionnaire were applied: Level 0: No dangerous behaviors; Level 1: verbal threats and shouting, but not smashing. Level 2: smashing and destroying family property but could be persuaded. Level 3: destroyed property regardless of circumstances and persuasion. Level 4: repeatedly smashing property or person regardless of circumstances and persuasion. Level 5: ranked top one in danger degree, armed violence against people, committed arson or triggered an explosion.

Above all, the following content would be obtained: 1). General demographic characteristics of respondents aged 15 years or older living in both the urban and rural areas; 2) 1-month weight prevalence and lifetime prevalence rate of SMI. 1-month prevalence estimate was perceived approximately as time-point prevalence since the respondent was requested to narrate mental status in a 1 month at the time they were interviewed. Lifetime prevalence was estimated based on historic illness assessing by SCID; 3) Geographical distribution and other distribution characteristics of prevalence in different age, gender, community, occupational category, educational level and marital status; 4) Public health consequences of SMI and health service utilization of patients with SMI.

### Quality control and training

Based on the informationalized survey system, quality control (QC) was implemented not only in the grass-roots interviewing field, but also across provincial and municipal level. QC supervisors were responsible for data verification, scene taping verification, telephone verification, scene photographs feedback, GPS locating the interviewer and scene-returning verification. 490 experts as the interviewers in the first-stage survey received three-day face-to-face training. The training content aimed at achieving the following goals: Firstly, trainees understand general investigation scheme, improve investigation techniques and know very well questionnaires content. Secondly, trainees learn about CAI methods and measures. Eventually, trainees need to take an examination to check consistency of survey results after the survey process rehearsal. 200 psychiatrists of second-stage survey were trained face-to-face for one day before the main survey started. The training content mainly focused on the epidemiological diagnostic tool of mental disorders, SCID-I. Besides interviewers, appropriately 60 supervisors received face-to-face training for preparing the solutions to problems that may occur in survey scene.

### Statistical analysis

Descriptive statistics were calculated for all variables. Categorical variables are summarized as frequencies and percentages. Chi square test was used to determine the differences of categorical variables between groups. Prevalence estimates were provided and expressed in absolute numbers and percentages with 95% confidence intervals (95% CIs). Age and gender weighted prevalence was estimated by standardization to the 2014 Hunan population by using Logistic regression analysis. The socio-demographic variables that may associate with SMI were also analyzed using Logistic regression analysis. All data including gender, age, urbanicity of residence, education level, employment status, and marital status were included in a multivariate logistic regression model. Odds ratios (ORs) and their 95% CIs were estimated using maximum likelihood methods. A two-side *P* value of < 0.05 was considered to be statistically significant. All analysis was performed with SPSS software, version 19.0 (SPSS Inc., Chicago, IL, USA).

## Results

### Socio-demographic characteristics of the participants

A total of 36900 households were recruited, and 35530 completed the survey, with a response rate of 96.3%. In the final stage of sampling, 89465 permanent residents aged 15 years or older were investigated, and 81.6% (72999) completed the screening process. Of the 72999 respondents, 56.0% aged from 30 to 59 years old, 50.4% were females, 50.6% lived in rural communities, 44.0% were farmers, and 82.1% were married. About one-third had education for 7–9 years and one-third for 1–6 years. (Table 1)

**Table 1:** Socio-demographic characteristics of participants (n = 72999).

Variables	No. (%)	SMI patients (n)	Weighted prevalence‰ (95% CI)	*P* value
**Age (years)**				0.069
15–29	9995 (13.7)	105	9.70 (6.74–13.94)	
30–44	17056 (23.4)	217	11.90 (9.30–15.21)	
45–59	23776 (32.6)	222	9.72 (7.60–12.43)	
60–64	7470 (10.2)	59	8.93 (5.18–15.34)	
≥65	14318 (19.6)	98	6.84 (6.24–7.44)	
**Gender**				0.214
Male	36185 (49.6)	331	10.16 (8.35–12.35)	
Female	36814 (50.4)	389	8.58 (7.09–10.39)	
**Community**				0.097
Urban	36030 (49.4)	330	8.61 (7.00–10.58)	
Rural	36969 (50.6)	390	10.74 (9.17–12.58)	
Education completed (years)^*^				0.033
Illiterate	5902 (8.1)	234	16.95 (11.53–24.85)	
Primary school	22660 (31.0)	362	10.16 (8.06–12.80)	
Junior high school	24931 (34.2)	64	9.31 (7.02–12.34)	
Senior high school/Technical secondary school	12261 (16.8)	13	8.95 (7.05–11.36)	
Tertiary, bachelor’s degree or above	6834 (9.4)	47	4.38 (2.72–7.07)	
Occupation^*^				< 0.001
Technology professionals/administrators	7819 (10.7)	22	3.04 (1.43–3.58)	
Industrial and commercial individual businessman	10263 (14.1)	36	3.93 (2.79–5.55)	
Farmers	32806 (44.0)	319	8.73 (6.80–10.54)	
Retirees	6708 (9.2)	40	6.90 (4.56–10.44)	
Jobless/unemployed	14222 (19.5)	287	18.22 (14.05–23.15)	
Marital status*				< 0.001
Unmarried	7060 (9.7)	171	22.69 (17.54–29.30)	
Married	59951 (82.1)	463	7.13 (5.89–8.64)	
Divorced	1010 (1.4)	36	28.61 (18.71–43.52)	
Widowed	4903 (6.7)	50	11.34 (7.27–17.65)	

CI, confidence interval. Referred to occupation, 1181/72999 respondents were not responded.

**Fig 2:**
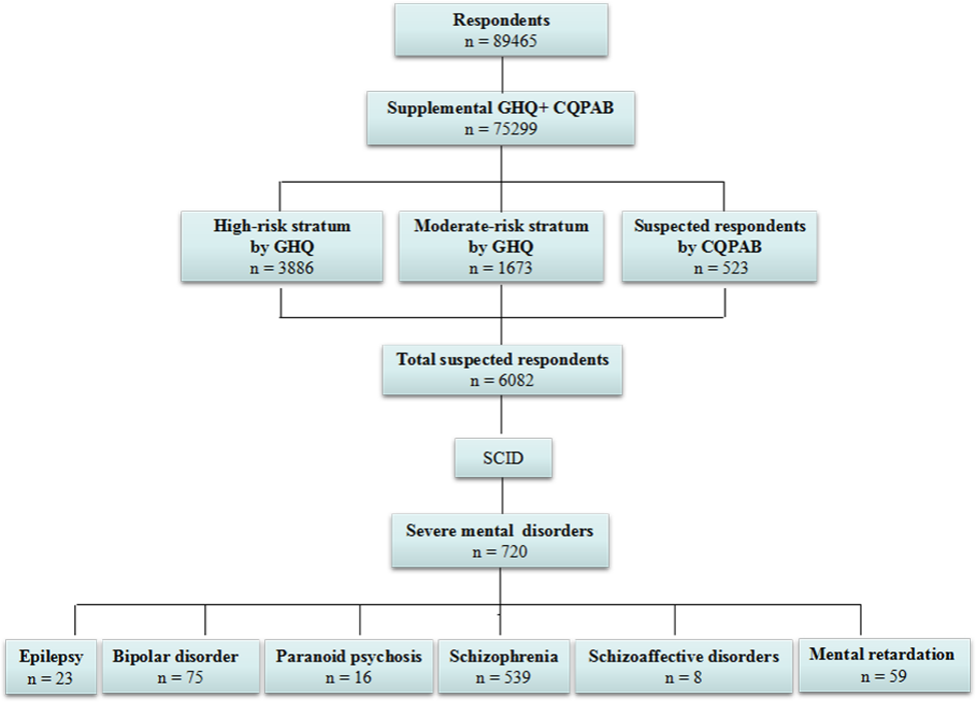
Flowchart showing permanent residents aged 15 years or older flowed in the two-phase survey in Hunan province. GHQ-12, the supplemental 12-Item General Health Questionnaire; CQPAB, Cue questionnaire of psychotic abnormal behaviors.

Among the 72999 respondents, 62587 answered GHQ-12 by themselves, 12765 answered by family members (Fig 2). The suspected respondents were synchronously judged by other reported CQPAB. High-risk stratum included 3886 respondents, with having GHQ score ≥ 4 and having high risk factors. Moderate-risk stratum included 1673 respondents with GHQ score of 2 or 3. The low-risk stratum which covered the remaining respondents with GHQ = 0–1 was terminated for interview. 523 suspected respondents with abnormal behaviors were identified according to CQPAB. The 6082 individuals were then assigned to complete the second-stage diagnostic assessment with SCID. Among them, 51.3% were females, 55.7% lived in rural communities, 49.3% aged from 30 to 59 years old (S1 Table). 79.2% of the 6082 respondents stated that they did not have mental and psychological problems, and 35.0% of the 6082 respondents stated that they had hospitalized due to physical diseases.

### 1-month and lifetime prevalence of SMI

720 individuals were definitely diagnosed as SMI (Fig 2). The overall 1-month and lifetime prevalence of SMI were 9.35‰ (8.13–10.75) and 10.10‰ (8.78–11.62), respectively (Table 2). The most frequently observed SMI was schizophrenia (n = 539), followed by bipolar disorder (n = 75), intellectual disability (n = 59), epileptic mental disorder (n = 23), paranoid psychosis (n = 16), and schizoaffective disorders (n = 8). Forty-one respondents reported more than one disorder. The 1-month prevalence of the six kinds of SMI ranged from 0.11‰ (0.03–0.32) to 6.50‰ (5.66–7.45), and the lifetime prevalence of the six kinds of SMI ranged from 0.24‰ (0.10–0.59) to 6.86‰ (5.99–7.87).

**Table 2:** The prevalence rate of different severe mental illness in Hunan Province (n = 72999).

SMI	N	Time-point prevalence rate (‰)	Lifetime prevalence rate (‰)
		(95% CI)	(95% CI)
Schizophrenia	539	6.50 (5.66–7.45)	6.86 (5.99–7.87)
Paranoid psychosis	16	0.25 (0.12–0.54)	0.26 (0.13–0.54)
Bipolar disorder	75	1.28 (0.75–2.18)	1.49 (0.94–2.36)
Schizoaffective disorders	8	0.11 (0.03–0.32)	0.24 (0.10–0.59)
Epileptic mental disorder	23	0.45 (0.25–0.80)	0.45 (0.26–0.80)
Intellectual disability	59	0.77 (0.48–1.23)	0.79 (0.50–1.25)
**Total**	720	9.35 (8.13–10.75)	10.10 (8.78, 11.62)

CI, confidence interval. Prevalence estimates are provided and expressed in percentages with 95% confidence intervals.

**Fig 3:**
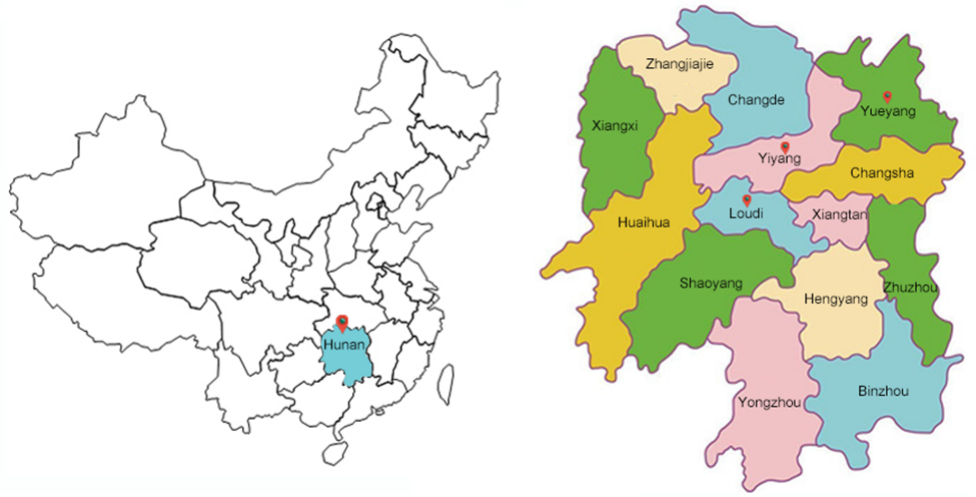
Geographical distribution maps of 14 cities surveyed in Hunan province.

As for the geographical distribution, the 1-month prevalence of SMI existed significant differences among 14 cities in Hunan Province (P = 0.004, Table 3 and Fig 3). The highest weighted prevalence was observed in Loudi (16.37‰, 95% CI: 12.80–20.90), followed by Yiyang (14.13‰, 95% CI: 8.47–23.47) and Yueyang (13.56‰, 95% CI: 10.40–17.65). The lowest weighted prevalence was observed in Changde (7.17‰, 95% CI: 4.94–10.41), Huaihua (7.05‰, 95% CI: 4.79–10.39), Xiangtan (4.48‰, 95% CI: 2.29–8.76).

**Table 3:** The geographical distribution of 1-month prevalence rate of severe mental illness in different cities in Hunan Province (n = 72999).

City	Total		Urban area		Rural area	
	N	Weighted prevalence (‰) (95% CI)	N	Weighted prevalence (‰) (95% CI)	N	Weighted prevalence (‰) (95% CI)
**Changde**	5435	7.17 (4.94–10.41)	2621	6.48 (4.37–9.61)	2814	8.58 (5.08–14.45)
**Binzhou**	5529	8.11 (4.98–13.18)	2747	7.77 (4.47–13.50)	2782	9.21 (3.97–21.22)
**Hengyang**	7587	10.41 (6.07–17.78)	3407	9.03 (4.69–17.30)	4180	15.05 (9.05–24.93)
**Huaihua**	8041	7.05 (4.79–10.39)	3850	6.34 (3.02–13.27)	4191	8.55 (7.50–9.76)
**Loudi**	3001	16.37 (12.80–20.90)	1490	16.20 (12.28–21.34)	1511	17.53 (13.07–23.47)
**Shaoyang**	5875	7.84 (4.97–12.35)	2921	5.61 (3.72–8.47)	2954	15.45 (10.87–21.93)
**Xiangtan**	2511	4.48 (2.29–8.76)	1124	1.91 (0.39–9.26)	1387	6.27 (5.01–7.85)
**Xiangxizhou**	5021	7.75 (5.46–10.99)	2471	6.22 (5.29–7.30)	2550	9.40 (5.85–15.08)
**Yiyang**	3664	14.13 (8.47–23.47)	1617	19.32 (11.54–32.15)	2047	10.17 (8.14–12.69)
**Yongzhou**	5299	7.59 (4.98–11.53)	2501	7.40 (3.74–14.62)	2798	8.02 (4.79–13.40)
**Yueyang**	6181	13.56 (10.40–17.65)	3028	15.87 (11.45–21.96)	3153	9.48 (7.89–11.40)
**Zhangjiajie**	2406	9.46 (5.15–17.31)	1250	13.81 (10.00–19.04)	1156	5.94 (4.83–7.30)
**Changsha**	6650	10.43 (7.15–15.18)	4231	6.42 (5.43–7.58)	2419	15.01 (13.83–16.28)
**Zhuzhou**	5799	7.92 (4.56–13.73)	2772	2.29 (1.44–3.65)	3027	12.66 (10.33–15.51)

CI, confidence interval. Weighted prevalence are provided and expressed in percentages with 95% confidence intervals.

### Socio-demographic characteristics related to 1-month prevalence of severe mental illness

Multivariate regression analysis indicated that lower education (literate, OR = 4.71, 95% CI: 2.99–7.43; 1–6 years, OR = 2.85, 95% CI: 1.87–4.33; 7–9 years, OR = 1.66, 95% CI: 1.11–2.50; 10–12 years, OR = 1.62, 95% CI: 1.08–2.45), farmer occupation (OR = 2.65, 95% CI: 1.66–4.22), retirees (OR = 3.27, 95% CI: 1.88–5.70) or jobless/unemployed (OR = 5.91, 95% CI: 3.72–9.40), unmarried (OR = 4.79, 95% CI: 3.20–7.16) or divorced (OR = 4.13, 95% CI: 2.58–6.60), and age of 30–44 years old (OR = 3.68, 95% CI: 2.72–4.96), 45–59 years old (OR = 2.34, 95% CI: 1.69–3.25) or 60–64 years old (OR = 1.48, 95% CI: 0.98–2.24) were found to be significantly associated with SMI (Table 4).

**Table 4:** Socio-demographic correlation of 1-month prevalence rate of severe mental illness.

Parameters	OR (95% CI)	*P*
**Constant variable**		< 0.01
**Gender (reference to Female)**		
Male	1.13 (0.96–1.34)	0.16
**Community (reference to Urban area)**		
Rural area	1.10 (0.93–1.29)	0.28
**Age (years) (reference to 15–29 age group)**		
30–44	3.68 (2.72–4.96)	< 0.01
45–59	2.34 (1.69–3.25)	< 0.01
60–64	1.48 (0.98–2.24)	0.06
> 65	1.27 (0.86–1.89)	0.23
**Education completed (years) (reference to tertiary degree or above)**		
Literate	4.71 (2.99–7.43)	< 0.01
Primary school	2.85 (1.87–4.33)	< 0.01
Junior high school	1.66 (1.11–2.50)	0.01
Senior high school/Technical secondary school	1.62 (1.08–2.45)	0.02
**Occupation (reference to technology professionals/administrators)**		
Industrial and commercial individual businessman	1.08 (0.63–1.86)	0.79
Farmers	2.65 (1.66–4.22)	< 0.01
Retirees	3.27 (1.88–5.70)	< 0.01
Jobless/unemployed	5.91 (3.72–9.40)	< 0.01
**Marital status (reference to windowed)**		
Unmarried	4.79 (3.20–7.16)	< 0.01
Married	0.92 (0.67–1.27)	0.62
Divorced	4.13 (2.58–6.60)	< 0.01

### Family and social impact of severe mental illness

**Fig 4:**
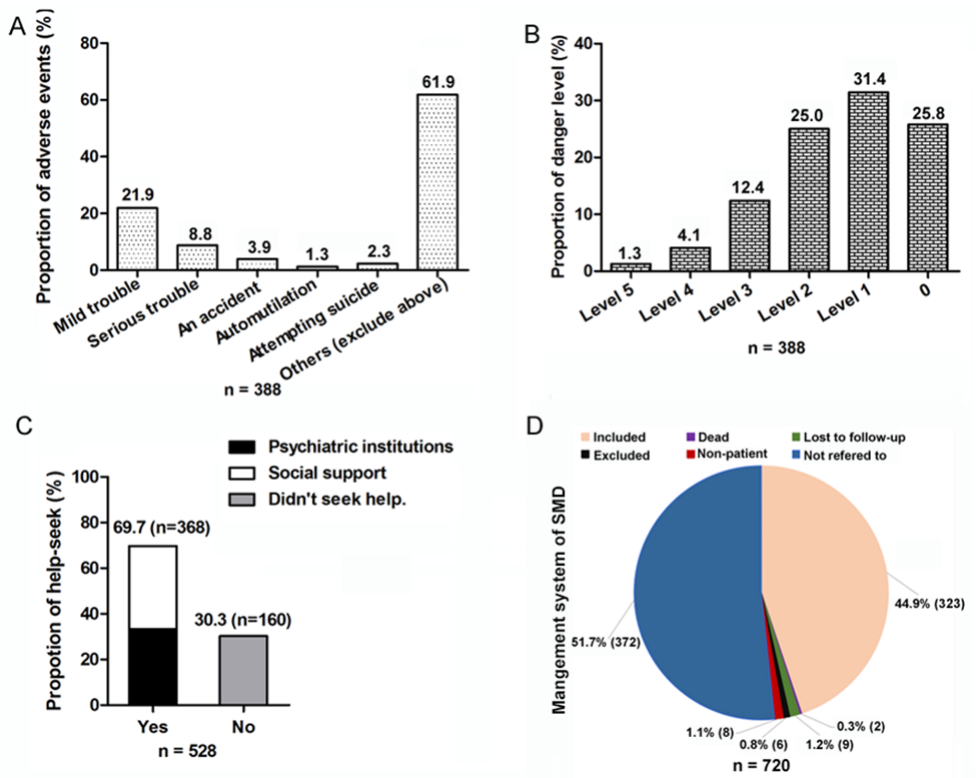
Impact and management of severe mental illness (SMI) in Hunan province. Panel A. Adverse events towards family and society. 388 of 720 patients with SMI completed the impact on family and society questionnaire and the danger assessment questionnaire. Panel B. Danger level of psychiatric diseases. 388 of 720 patients with SMI completed the danger assessment questionnaire. 1–5 represents the different levels. Panel C. Help-seek behaviors of patients. 528 of 720 patients with SMI completed the help-seeking behavior questionnaire and medical service utilization questionnaire. Social support represented that patients seek help from individual doctor, relatives, colleagues, friends, neighbors, witch doctor, invoking God or the Buddha. Psychiatric institution represented that patients seek consultation or treatment via outpatient or hospitalization in specialized medical institutions for psychosis. Panel D. The management status of patients included or excluded from SMI management system of Hunan province.

Of the 720 SMI patients, 388 completed the impact on family and society questionnaire and the danger assessment questionnaire. The impact assessment revealed an adverse event rate of 38.1% (147/388, Fig 4A). Mild trouble (21.9%) was the common adverse event, followed by serious trouble (8.8%), accident (3.9%), attempting suicide (2.3%) and automultilation (1.3%). The danger assessment questionnaire showed that 74.2% of the 388 individuals had danger behaviors from Level 1 to 5 (Fig 4B).

### The help-seeking behaviors, utilization of mental health services, and management status of patients with severe mental illness

Of the 720 individuals with SMI, 528 completed the help-seeking behavior questionnaire and medical service utilization questionnaire. 69.7% (368) of the 528 patients had sought help (Fig 4C), with detailed data shown in S2 Table. Of the 528 patients, 33.3% sought help via outpatient or hospitalization in specialized medical institutions for psychosis, and 36.4% sought for a variety of social supports, including individual doctor, families and relatives, colleagues and friends, neighbors, witch doctor, God or the Buddha.

The reasons that patients did not seek help were shown in S3 Table. The following reasons ranked top three: 47.4% of the 528 patients concerned about too high cost. 39.6% of patients considered their diseases as mild, and thought there was no need to seek help. 34.3% minded others knowing their mental illness.

Of the 720 individuals with SMI, a total of 348 (48.3%) patients were recorded in the SMI management system of Hunan province. As for the 48.3% respondents, 44.9% was still included in the system, 0.3% was dead at the time of data collection, 1.2% was lost to follow up, 0.8% was excluded from the system, and 1.1% was non-patient in the system. However, 51.7% of the patients were not referred to the management system which were not explained (Fig 4D).

## Discussion

The mortality of SMI, including schizophrenia, bipolar disorder, schizoaffective disorder etc., was two or three times as high as that of the general population [32–36]. Given increasing public health concerns of SMI, a clear epidemiologically based dataset was essential. Only a large survey of psychiatric disorders was performed in a representative sample of 9,495 children (aged 5–17 years older) in Hunan province, and it mainly focused on attention-deficit disorders and disruptive behavior disorders [37]. Therefore, a large-scale epidemiological survey was performed in Hunan province to obtain the prevalence estimate, distribution characteristics and current management status of SMI. Our survey indicated that the most common SMI was schizophrenia, followed by bipolar disorder, intellectual disability, epileptic mental disorder, paranoid psychosis and schizoaffective disorders. Several socio-demographic characteristics were associated with increased risk for SMI. We found that SMI brought different levels of danger and adverse event. However, the proportion of help-seek behaviors was not very high, and more than a half of the patients were not referred to the management system of SMI. These findings provided the comprehensive recognition of SMI in Hunan province and the basis for making rational treatment measure and public health policy.

Previous survey reported that psychiatric disorders were inclined to emerge in late adolescence and young adulthood [38]. Thus, our investigation focused on the residents aged 15 years or older in in Hunan province. A total of 89465 individuals were randomly selected from the 123 counties and districts of 14 cities. Compared with previous national epidemiological survey in 4 provinces in China, individuals aged 18 years or older were selected as sampling frame [14], thus it indicated that our sample coverage was more representative. Here, up to 81.6% of the respondents completed GHQ-12. After first-stage initial screening to confirm 6082 suspected respondents, less than 10% of the 72999 respondents received the second-stage diagnosis, this greatly reduced the workload of interviewers since SCID-I need to be administered merely by psychiatrists. The data indicated that 1-month and lifetime weight prevalence of SMI in Hunan province were 9.35‰ and 10.10‰, which represented the proportion of individuals who manifested a disorder at a given point in 1 month and the proportion of individuals in the population who have ever manifested a disorder, respectively. The prevalence data here was gathered during one month. As previous survey indicated, the short time spent at each primary sampling site would increase inter-rater reliability of the SCID and increase our estimates of reliability [14].

In our survey, schizophrenia had the 1-month and lifetime prevalence of 6.50‰ and 6.86‰, ranking top one. According to the across-national survey based on four provinces in China during 2001–2005, the adjusted prevalence of schizophrenia was 7.81‰ [14]. Another across-provincial survey in Hebei in China during 2004–2005 reported that the prevalence of schizophrenia was 5.46‰ [16]. As for bipolar disorder, our 1-month and lifetime prevalence estimate was 1.28‰ and 1.49‰, higher than the adjusted 1-month prevalence of 0.99‰ (bipolar I disorder), 0.26‰ (bipolar II disorder) reported in the survey of four provinces in China [14]. And the time-point and lifetime prevalence of bipolar I/II disorder were 1.25‰ and 1.97‰/0.48‰ and 1.30‰ reported in the survey of Hebei province, respectively [16]. In addition, our 1-month and lifetime prevalence estimate of schizoaffective disorders was estimated as 0.11‰ and 0.24‰, lower than 0.24‰ and 0.47‰ reported in the survey of Hebei province [16], and slightly lower than adjusted prevalence of 0.20‰ reported in the survey of four provinces in China [14]. Ding D *et*.*al* mentioned that several epidemiologic surveys of epilepsy carried out in China, the lifetime prevalence of epilepsy was 3‰-5‰, and the incidence of epilepsy was 0.3–0.4‰ [39]. Our 1-month and lifetime prevalence estimate of epileptic mental disorder were 0.45‰ and 0.45‰. Above all, the increase or decrease in the prevalence of any SMI was found when compared with previous surveys. Dinesh Bhugra *et*.*al* reported that the prevalence estimates of schizophrenia would differ between lifetime, period, and point prevalence [40]. In addition, it may attribute to the distinctions of sampling method, field quality control, diagnostic criteria and investigation procedure etc..

As for geographical distribution of prevalence, six kinds of severe psychiatric disorders in 14 cities, especially exist distinctions in urban and rural area, which may be related to a variety of economy and education level etc.. In our survey, the prevalence of SMI was higher in rural *versus* urban area [10.74‰ (9.17–12.58) *versus* 8.61‰ (7.00–10.58)], without significant difference. Logistic regression analysis showed that community (rural or urban area) could not be considered as the risk factor of SMI, so did and gender (male or female). The prevalence of SMI was higher in male versus female [10.16‰ (8.35–12.35) versus 8.58‰ (7.09–10.39)], without significant difference. The previous epidemiological survey of 4 provinces in China analogously showed that the prevalence of schizophrenia showed no significant difference in gender and community distribution, not like major depressive disorder, dysthymia disorder, alcohol dependence [14]. Numbers of large-scale mental health surveys globally providing population prevalence estimates similarly indicated that compared in women and man, mood (7.3%:4.0%), anxiety (8.7%:4.3%) disorders and substance use disorders (2.0%:7.5%) showed different high risk [41]. In our survey, the illiterate (OR = 4.7, 95% CI: 2.99–7.43), jobless/unemployed (OR = 5.9, 95% CI: 3.72–9.40), unmarried (OR = 4.8, 95% CI: 3.20–7.16) and divorced individuals (OR = 4.1, 95% CI: 2.58–6.60) had much higher risk for SMI. As an epidemiologic survey among 18571 people in the United States reported that the separated or divorced people have higher risk for mental disorders than the married people [42]. Cross-national epidemiological surveys of mental disorders indicated that the highest estimated prevalence was mainly found among respondents at the lowest level of educational attainment [11]. In addition, respondents in the age group of 30–44 years (OR = 3.68, 11.9‰, 95% CI: 9.30–15.21) and 45–59 years (OR = 2.34, 9.72‰, 95% CI: 7.60–12.43) had higher risk for SMI *versus* the age group of 15–29 years (OR = 9.70‰, 6.74–13.94). Above all, these socio-demographic distribution characteristics revealed that literacy, unemployment, unmarried/single, divorce and middle-age were associated with increased risk of SMI.

Literatures reported evidences that people with mental illness brought danger to themselves or others [43, 44]. For example, Lynne Jones *et al*. mentioned a patient with paranoid psychosis was aggressive, thus restrained by his family, but further deteriorated, resulting from inadequate shelter and absence of appropriate care [45]. Our survey indicated that total 3.6% of 388 patients attempted automutilation or suicide, 34.5% of 388 patients caused trouble or an accident. And 68.8% of 388 patients had behaviors of threats and shouting, hitting, destroying property (Level 1–3). More seriously, 5.4% of 388 patients caused threaten to others’ property and personal security (Level 4 and 5). SMI could bring enormous burden to family and society in both urban and rural community. Our epidemiological work strengthened the extent of the problem due to severe psychiatric disorders.

The survey here indicated 368 individuals really seek help in 528 patients with SMI, who completed the seek-help behavior questionnaire, the ratio of seeking help from social support (n = 176) *versus* psychiatric institutions (n = 192) was nearly 1:1 (33.3%:36:4%). In addition, 30.3% (n = 160) patients never seek help. Thus, the majority of the patients with SMI did not receive professional therapy or were not treated very well. Just as a previous study reported, there was unmet treatment needs even in the two most developed cities (Beijing and Shanghai) [46]. It reported that stigma, low mental health knowledge and perceived need, and high treatment fees constituted the barriers to mental health service utilization, just as our survey concluded. What’s more, according to the data here, 51.67% of 720 patients were not involved in the management system of SMI in Hunan province, which suggested the unavailability of mental health service. A cross-sectional, nationally representative household survey in United States similarly proposed unmet need for treatment of SMI due to personal insensibility, situational barriers and financial barriers [13]. A previous study around six European countries indicated that more available resources do not always result in greater use of services for people with mental disorders [47]. Thus, self-recognition of need for treatment and effective patient centeredness of cares should be strengthened.

This study also has some limitations. Primarily, a random sample of first-stage negative subjects was not performed in the second stage interview due to an extremely large number of subjects involved and an inadequate supply of physicians. It would inevitably and unavoidably cause the missed diagnosis or under estimation in our study. Besides, we did not perform stratified analysis whether community, gender and age range, marriage, occupation could be risk factors for each SMI. Nevertheless, the findings of our study did provide some information on the prevalence, distribution, impact, and management of SMI in Hunan Province, China, which may provide evidence for better assignment of the mental health resources.

## Conclusions

In conclusion, our survey is the first large-scale epidemiological survey of severe mental illness for Chinese population in Hunan province. The advantage of this survey lied in the large sample population, rigorous quality control, widely-used diagnostic criteria and strict investigation procedures etc.. Importantly, the information about prevalence, distribution, impact and danger, and management may provide evidence for assigning mental health resources.

## Supporting information

S1 Table Socio-demographic characteristics of respondents completed two-phase survey in Hunan Province (n = 6082). (DOC)

S2 Table The utilization of mental health services for individuals with severe mental illness in Hunan Province. Note: 528 of 720 patients with SMI completed the help-seeking behavior questionnaire.
(DOC)

S3 Table A variety of reasons which explained that patients with severe mental illness did not seek help in Hunan province (n = 528). Note: 528 of 720 individuals with severe mental illness completed the health services assessment questionnaire.
(DOC)
