# Supplementary material for: Epidemiology of severe mental illness in Hunan province in central China during 2014-2015: A multistage cross-sectional study
Source: PLoS One. 2017 Nov 29;12(11):e0188312. doi: 10.1371/journal.pone.0188312 (PMC5706681; doi:10.1371/journal.pone.0188312)
Supplement: S1 Table — (DOC) [file pone.0188312.s001.doc]

**Supplemental Table 1** Socio-demographic characteristics of respondents completed two-phase survey in Hunan Province (n = 6082)

| Variables | No. (%) |
| --- | --- |
| Age (years) |  |
| 15-30 | 603 (9.9) |
| 30-44 | 996 (16.4) |
| 45-59 | 2000 (32.9) |
| 60-64 | 700 (11.5) |
| >65 | 1604 (26.3) |
| other | 179 (3.0) |
| Gender |  |
| Male | 2960 (48.7) |
| Female | 3122 (51.3) |
| Community |  |
| Urban | 2758 (45.3) |
| Rural | 3314 (55.7) |
| Education completed (years) |  |
| 0 (Literate) | 1174 (19.3) |
| 1-6 Primary school) | 2186 (35.9) |
| 7-9 (Junior high school)） | 1574 (25.9) |
| 10-12([Senior](../../../../C:/Users/YL807/AppData/Local/Yodao/DeskDict/frame/20160129143038/javascript:void(0)%3B) [high](../../../../C:/Users/YL807/AppData/Local/Yodao/DeskDict/frame/20160129143038/javascript:void(0)%3B) [school](../../../../C:/Users/YL807/AppData/Local/Yodao/DeskDict/frame/20160129143038/javascript:void(0)%3B)/Technical secondary school) | 603 (9.9) |
| 13-22 (Tertiary degree , bachelor’s degree or above) | 333 (5.5) |
| Unknown | 117 (19.2) |
| Marital status |  |
| Married and cohabitation | 4246 (69.8) |
| Divorced and widowed | 972 (16.0) |
| Unmarried | 702 (11.5) |
| Others | 162 (2.7) |
| Occupation |  |
| Farmer, worker and individual business/ temporary workers | 3359 (55.2) |
| Students/housewives,technology professionals/administrators | 419 (6.9) |
| Jobless / unemployed | 1013 (16.6) |
| Others | 762 (12.5) |
| Whether or not you have mental and psychological problems? |  |
| No | 4815 (79.2) |
| Yes | 688 (11.3) |
| Others | 579 (9.5) |
| How often do you drink? |  |
| Don't drink | 4321 (71.0) |
| Occasionally a small amount of  alcohol | 565 (9.3) |
| Regular a small amount of  alcohol | 330 (5.4) |
| Often a large number of  alcohol | 191 (3.1) |
| Others | 675 (11.1) |
| Whether or not you have been in hospitalization due to physical diseases? |  |
| No | 3375 (55.5) |
| Yes | 2130 (35.0) |
| Others | 577 (9.5) |
