# Supplementary material for: Epidemiology of severe mental illness in Hunan province in central China during 2014-2015: A multistage cross-sectional study
Source: PLoS One. 2017 Nov 29;12(11):e0188312. doi: 10.1371/journal.pone.0188312 (PMC5706681; doi:10.1371/journal.pone.0188312)
Supplement: S2 Table — Note: 528 of 720 patients with SMI completed the help-seeking behavior questionnaire. (DOC) [file pone.0188312.s002.doc]

**Supplemental Table 2** The utilization of mental health services for individuals with severe mental disorders in Hunan Province

| The models of help-seeking for patients | No. |
| --- | --- |
| Psychiatric Institutions | 176 (33.3%) |
| Hospitalization in a psychiatric hospital | 125 |
| Outpatient in a psychiatric hospital | 49 |
| Consultation outpatient in a psychiatric hospital | 2 |
| Social Support | 192 (36.4%) |
| Outpatient in the psychiatric departments of a general hospital | 31 |
| Outpatient in the internal medicine department of a general hospital | 9 |
| Network consultation | 3 |
| Community mental institution | 1 |
| Hospitalization in a general hospital | 1 |
| Individual doctor | 8 |
| Community Pharmacy | 2 |
| Families and relatives | 61 |
| Colleagues and friends | 6 |
| Witch doctor, invoking God or the Buddha and qigong etc. | 42 |
| Other non-medical services institutions and individuals | 28 |

Note: 528 of 720 patients with SMD completed the help-seeking behavior questionnaire
