# Supplementary material for: Epidemiology of severe mental illness in Hunan province in central China during 2014-2015: A multistage cross-sectional study
Source: PLoS One. 2017 Nov 29;12(11):e0188312. doi: 10.1371/journal.pone.0188312 (PMC5706681; doi:10.1371/journal.pone.0188312)
Supplement: S3 Table — Note: 528 of 720 individuals with severe mental illness completed the health services assessment questionnaire. (DOC) [file pone.0188312.s003.doc]

**Supplemental Table 3** A variety of reasons which explained that patients with severe mental disorders did not seek help in Hunan province (n = 528).

| Reasons that patients did not seek help | Yes (%) | No (%) | Unknown/Refuse to answer (%) | Other reasons (%) |
| --- | --- | --- | --- | --- |
| I am worried that I could not afford too high cost. | 250 (47.4) | 227 (43.0) | 48 (9.1) | 3 (0.6) |
| I consider it as mild disease, thus there is no need to seek help. | 209 (39.6) | 263 (49.8) | 54 (10.2) | 2 (0.4) |
| I am afraid that others knew I had mental illness. | 181 (34.3) | 295 (55.9) | 51 (9.7) | 1 (0.2) |
| I care about others’ attitude when I was found to receive psychiatric therapy. | 169 (32.0) | 301 (57.0) | 57 (10.8) | 1 (0.2) |
| I do not think the treatment will work out obviously. | 167 (31.6) | 312 (59.1) | 46 (8.7) | 3 (0.6) |
| I am afraid that I will be forced to be in hospital against my will. | 165 (31.3) | 308 (58.3) | 54 (10.2) | 1 (0.2) |
| I do not know where to seek the treatment or who I can turn to for help. | 157 (29.7) | 311 (58.9) | 57 (10.8) | 3 (0.6) |
| I want to figure out this problem by myself. | 131 (24.8) | 333 (63.1) | 60 (11.4) | 4 (0.8) |
| I think it is a waste of time or it is inconvenient. | 123 (23.3) | 348 (65.9) | 54 (10.2) | 3 (0.6) |
| I have other difficulties so that I can hardly receive the treatment. | 120 (22.7) | 348 (65.9) | 57 (10.8) | 3 (0.6) |
| I am unsatisfactory with the current medical service. | 51 (9.7) | 416 (78.8) | 58 (11.0) | 3 (0.6) |
| I don't have medical insurance. | 28 (5.3) | 451 (85.4) | 46 (8.7) | 3 (0.6) |

Note: 528 of 720 individuals with severe mental disorders completed the health services assessment questionnaire.
